# Medication Adherence in Vietnamese Patients with Cardiovascular and Endocrine–Metabolic Diseases

**DOI:** 10.3390/healthcare10091734

**Published:** 2022-09-09

**Authors:** Hung Huynh Vinh Ly, Ngoc Nguyen Minh Le, Mai Thi Thao Ha, Han Gia Diep, Anh Nhut Lam, Thao Thi Thanh Nguyen, Duyen Thi Nhan Le, Trang Thi Nhu Nguyen, Tu Thi Cam Le, Katja Taxis, Suol Thanh Pham, Khanh Duy Dang, Thang Nguyen

**Affiliations:** 1 Faculty of Medicine, Can Tho University of Medicine and Pharmacy, Can Tho City 900000, Vietnam; 2 Department of Traditional Medicine, Can Tho University of Medicine and Pharmacy, Can Tho City 900000, Vietnam; 3 Department of Pharmacology and Clinical Pharmacy, Can Tho University of Medicine and Pharmacy, Can Tho City 900000, Vietnam; 4 Faculty of Public Health, Can Tho University of Medicine and Pharmacy, Can Tho City 900000, Vietnam; 5 Office of Science and Technology—External Relations, Can Tho University of Medicine and Pharmacy, Can Tho City 900000, Vietnam; 6 Groningen Research Institute of Pharmacy, University of Groningen, 9713 Groningen, The Netherlands

**Keywords:** medication adherence, cardiovascular and endocrine–metabolic diseases, COVID-19, Vietnamese

## Abstract

(1) Background: COVID-19 has significantly affected the quality of life and the medication adherence of patients with chronic diseases. Attitudes towards the disease and preventive measures are the things that need to be considered for patient adherence to medication during the COVID-19 pandemic. We aimed to evaluate the rate and compare the medication adherence and the impact of the COVID-19 pandemic on medication adherence in Vietnamese patients with cardiovascular and endocrine–metabolic diseases. (2) Methods: A cross-sectional study was conducted on outpatients having chronic diseases such as cardiovascular or/and endocrine–metabolic diseases in some southern provinces in Vietnam. In each group of patients, medication adherence was measured and assessed with the General Medication Adherence Scale (GMAS), adjusted and validated in Vietnam. In addition, the study also investigated attitudes and practices to prevent COVID-19. (3) Results: Out of 1444 patients in our study, the level of adherence was recorded in 867 cases, accounting for 61.1%. The group of patients with only cardiovascular disease and patients with only endocrine–metabolic disease had relatively similar compliance rates of 62 and 61.1%, respectively. The leading cause of non-adherence to treatment in all three groups of patients in the study, as assessed by the GMAS, was non-adherence due to financial constraints. Our study showed that 71.6% of patients felt anxious when going to the hospital for a medical examination. However, only 53.7% identified the COVID-19 pandemic as obstructing treatment follow-up visits. The research results showed that the COVID-19 epidemic influences the patient’s psychology with regard to re-examination and treatment adherence, with *p* coefficients of 0.003 and <0.001, respectively. (4) Conclusion: Medication adherence rates in two disease groups are close, and financial constraint is the fundamental reason for medication non-adherence. Regulatory agencies must take care of people’s welfare to improve adherence in the epidemic context.

## 1. Introduction

SARS-CoV-2 causing COVID-19 has been at pandemic levels since March 2020 [1]. It causes morbidity in the range of respiratory illness to severe complications characterized by acute respiratory distress syndrome and has a high mortality rate [2]. The fast-spreading and difficult-to-control COVID-19 has quickly led to the restriction of social interactions and the closure of several service facilities in many countries worldwide, including Vietnam [3], which has affected people’s psychology in general and patients who are being treated for chronic diseases [2,3]. According to the survey, many people are concerned about the COVID-19 epidemic, which affects the patient’s adherence to treatment during routine check-ups. A systematic review of COVID-19 patients showed that individuals with hypertension, diabetes, and cardiovascular and respiratory system diseases were the most vulnerable or at risk of fatality. These groups of patients are the groups that clinical doctors are concerned about, specifically with regard to their treatment results during the complicated development of the epidemic.

As the COVID-19 epidemic situation increases, the number of patients with chronic medical conditions hospitalized for COVID-19 is also remarkable. Non-adherence has significantly affected patients’ quality of health and their lives. Out of 1150 patients hospitalized with COVID-19, 82% had at least one chronic disease related to the cardiovascular (63%) and endocrine–metabolic (36%) system [4]. Some authors suggest that patients with cardiovascular disease before COVID-19 have a higher mortality rate, are more likely to develop severe disease, and require more ventilatory intervention than those without the concomitant cardiovascular disease [5,6,7]. In addition, more than 50% of patients with diabetes have a higher mortality rate than non-diabetic patients [6]. Due to long-term and continuous use of drugs to ensure that blood sugar does not exceed the threshold, many patients have unilaterally ceased or not utilized the doctor’s prescription, leading to several studies to show that about 50% of patients were not treated effectively, leaving many complications such as cardiovascular, neurological, eye, kidney, and feet complications [8,9]. Hypertension, diabetes, and cardiovascular diseases are common in the majority of patients with chronic diseases. It is a chronic ailment that needs lifetime pharmaceutical treatment, and the number of patients is rising yearly [8]. Factors such as age, disease awareness, monthly income, length of treatment, the burden of medication, and treatment costs often affect the patient’s adherence to medication, and COVID-19 is a barrier to adherence [10,11,12,13].

Patients with chronic diseases would have a reduced risk of complications if they followed treatment guidelines [13]. During the COVID-19 pandemic, attention should be paid to adherence to treatment to minimize the negative impact of the disease on these patients [6]. However, data on drug adherence in patients with chronic diseases, particularly cardiovascular and endocrine–metabolic diseases, during the COVID-19 epidemic period in Vietnam were limited. Therefore, we conducted a study with two objectives. The primary objective is to determine the rate and compare the level of medication adherence in patients with chronic cardiovascular and endocrine–metabolic diseases, and the secondary objective is to evaluate the influence of knowledge and attitudes about the COVID-19 pandemic on adherence. From the above objectives, the study will provide objective data to take specific actions that affect patient adherence to help clinicians better treat patients.

## 2. Materials and Methods

### 2.1. Setting and the Study Population

A cross-sectional study was conducted on outpatients having chronic diseases such as cardiovascular or/and endocrine–metabolic diseases in some southern provinces in Vietnam from March to May. The selection criteria were patients with chronic diseases who had been treated for 6 months or more and who had come to the outpatient clinics of hospitals at the study site. The study excluded the cases of patients who did not agree to participate in the study and patients who had a follow-up appointment but could not be contacted.

### 2.2. Data Sources and Method of Analysis

Sampling collaborators who interviewed the patients to collect the characteristics of the study subjects (their age group, gender, place of residence, occupation, education level, monthly income, duration of illness, duration of treatment, drug adherence, attitudes, and practices to prevent COVID-19) were pre-trained by the research team.

Patient diagnoses were based on the “International Classification of Diseases-Tenth Revision” (ICD-10) of the World Health Organization (WHO). These data were divided into two large groups, cardiovascular and endocrine–metabolic diseases. Cardiovascular diseases such as arterial hypertension and atherosclerosis belong to the cardiovascular group, and endocrine–metabolic diseases such as diabetes, hyperthyroidism, and hypothyroidism belong to the endocrine–metabolic group. In each group of patients, medication adherence was measured and assessed through the General Medication Adherence Scale (GMAS), which was adjusted and validated in Vietnam [14,15]. The GMAS contains 11 questions, scored from 0 to 3 points for each one, which surveys 3 aspects of drug adherence: (1) patient behavior (5 questions), (2) additional disease and pill burden (4 questions), and (3) financial constraints (2 questions). The total score of the scale fluctuates from 0 to 33 points, and is categorized into 5 levels: high (30–33 points), good (27–29 points), partial (17–26 points), low (11–16 points), and poor (0–11 points). From the total score (0–33 points), we classified it into two groups: non-adherence (0–26 points) and adherence (≥27 points). The proportion of medication adherence was determined by the number of compliant patients divided by the total number of patients at the study time.

The impact of the COVID-19 pandemic on medication adherence was assessed through attitude and practice. Patient perspective includes their opinions on the impacts of the pandemic, which drives them to waver in the continuation of their treatments and follow-up care. The patient practice was surveyed based on their adherence to the “5 ***K***-message” with wearing a facemask *(**K**hau trang)*, disinfection *(**K**hu khuan)*, keeping distance *(**K**hoang cach)*, not gathering *(**K**hong tap trung)*, and health declaration (***K****hai bao y te*). The combination of measures increases the effectiveness of pandemic prevention. Therefore, we also investigated the measure combination (0–1 measure and ≥2 measure group). The χ^2^ test, with odds ratios (OR) and 95% confidence intervals (CIs), was applied to analyze the data and assess the relationship between medication adherence and population characteristics and the impact of the COVID-19 pandemic.

Descriptive statistics were used to summarize categorical (proportions and frequencies) and continuous variables (means, standard deviations). The goodness of fit for logistic regression models was evaluated by the Hosmer–Lemeshow test. The data were calculated using SPSS version 22.0 (IBM Corp., New York, the United) and Microsoft Excel software. The outcomes were statistically significant if the *p*-values were ≤ 0.05. The variables with univariable results with *p*-values ≤ 0.1 were analyzed by multivariable logistic regression with the Backward Wald method. The comparison standard was based on the probability to confirm the data accuracy, which aimed to evaluate the impact of the variables on medication adherence.

### 2.3. Ethics

Ethical issues for this study were approved by the Scientific Review Committee of Can Tho University of Medicine and Pharmacy (approval number: 1541/QDDHYDCT in 2021). Participants were informed of the purpose and procedure of the study and voluntarily signed an informed consent form.

## 3. Results

Table 1 shows the demographics of the patients. After excluding non-standard samples, the study selected 1444 samples (female = 816; 56.5%). There were no statistically significant differences in gender, living places, employment, level of education, or monthly income among groups of patients. Additionally, there was a significant difference in the proportion of patients ≥ 60 years of age, the duration of disease, and treatment > 5 years between endocrine–metabolic disease and patients with both diseases (*p* < 0.001).

The GMAS for each group of patients is described in Table 2. The common adherence was relatively low (only about 50%, including high and good), and high adherence accounted for the highest proportion (534; 37.0%). Between the three groups of research subjects, the proportion of patients who complied with high, good, and partial adherence showed almost no difference. Low and poor adherence was often identified in the group with two diseases simultaneously rather than just one disease (cardiovascular or endocrine–metabolic disease). Expenditure on treatment was the most significant cause of non-adherence in three groups of research subjects, with the lowest ratio in patients of both diseases (71.3%), and it was a significant difference compared to the cardiovascular patients—the highest ratio group (81.4%) (*p* < 0.001).

Patient behavior, additional disease and pill burden, and financial constraints were the causes of non-adherence. The behavioral factor was the second most common cause of non-adherence, with a difference between the group of endocrine–metabolic patients and patients of both diseases compared to the cardiovascular patients (*p* < 0.05) (Table 3).

Most patients wore masks for COVID-19 epidemic prevention (95.2%). Table 4 showed that the implementation rate of epidemic measures according to the recommended 5 ***K*** of Vietnam’s Ministry of Health was almost similar between patient groups except for disinfection measures. Only 7% of the endocrine–metabolic patients performed disinfection measures, less than six times for the remaining two groups (44% in the cardiovascular patients and 47.5% in the endocrine–metabolic patients).

The mean number of times the patient performed an action to prevent the COVID-19-epidemic when they came to see the doctor was 1.92 ± 1.191. Specifically, the rate of five preventive measures taken by patients in each group, calculated on average in the cardiovascular, endocrine–metabolic, and both groups, was 1.91 ± 1.188, 1.89 ± 1.229, and 1.97 ± 1.152, respectively. In particular, the implementation of >1 measure to prevent COVID-19 in the endocrine–metabolic disease was the lowest. At the same time, the study also recorded 1034/1444 cases of patients who felt worried when coming to the hospital for follow-up visits during the epidemic. A total of 775 cases identified that the COVID-19 pandemic obstructed treatment follow-up visits.

We conducted a correlation to determine the effects of patient characteristics, such as age group (*p* = 0.048), job (*p* = 0.006), and comorbidities (*p* < 0.001), on adherence. At the same time, there was a correlation between adherence to measures to prevent COVID-19 (*p* < 0.001) and the effect of the COVID-19 pandemic (*p* < 0.001). (Table 5).

## 4. Discussion

### 4.1. Principal Findings

Out of the 1444 patients in our study, the level of adherence was recorded in 867 cases, accounting for 61.1%. The group of patients with only cardiovascular disease and patients with only endocrine–metabolic disease had relatively similar compliance rates of 62 and 61.1%, respectively. The leading cause of non-adherence to treatment in all three groups of patients in the study, as assessed by the GMAS, was non-adherence due to financial constraints. Our study showed that 71.6% of patients felt anxious when going to the hospital for a medical examination. However, only 53.7% identified the COVID-19 pandemic as a factor that obstructed treatment follow-up visits. The research results showed that the COVID-19 epidemic influences the patient’s psychology with regard to re-examination and treatment adherence, with *p* coefficients of 0.003 and <0.001, respectively.

### 4.2. Appraisal of Methods

The study used a cross-sectional descriptive method and conducted convenience sampling to investigate compliance, the influence of patient characteristics, and COVID-19 on drug adherence. The strength of this design is that the execution time is relatively fast, inexpensive, and there is no problem of losing track. Therefore, it is appropriate for the COVID-19 situation, when the implementation of contact restrictions to reduce the spread of the disease causes patients to refuse to participate in the study or easily lose track of it. We used the GMAS to assess the adherence to medication of patients with chronic diseases. In addition to determining the rate of drug adherence on this scale, we generally evaluate the causes of the patient’s non-adherence to treatment based on the influencing factors of cost, behavior, and comorbidities, thereby contributing to assessing the impact of COVID-19 on the economic and psychological life of patients undergoing treatment.

### 4.3. Possible Explanations and Comparisons with Other Studies

The patients with only cardiovascular or endocrine–metabolic disease had higher adherence to medication than patients with both diseases (Table 5). Amaia’s research also found that comorbidity was positively and significantly associated with non-adherence. Conversely, non-adherence was negatively and significantly related to cardiovascular risk factors and higher rates of annual general practitioner visits [4]. Gregory A Nichols also found that adherence was more common in patients with less than two comorbidities [16]. One of our studies mentioned that one of the causes of drug-related problems (DRPs) in patients is comorbidities and the number of drugs used in the prescription. The number of comorbidities is directly proportional to the negative psychology of the patient due to the prolonged treatment process, the increase in the number of drugs in the prescription, the number of times the patients take their medication, the cost of treatment, and the psychology of always facing bad progress when comorbidities are severe. Patients with psychological instability during treatment will have their compliance affected. It is easy for patients to forget instructions from their doctor or clinical pharmacist or to forget to take their medication several times a day. Psychological counseling for patients during the epidemic is also an issue that needs attention in the patient’s adherence.

In this study, 66.4% of patients said that the COVID-19 pandemic did not affect their adherence (*p* < 0.001) (Table 5). It can be explained that during the epidemic’s peak, Vietnam’s Ministry of Health allowed pharmacies to be operated by pharmacists and provided many free hotlines of doctors or clinical pharmacists so that patients could be consulted when their medication or health condition was not good. At the same time, implementing more than one COVID-19 prevention measure in hospitals and clinics has made people more confident when taking medication during the epidemic period (Table 4). The Vietnamese government always creates optimal conditions for patients during the epidemic period, including outbreak announcements and steering documents, medical measures, blockades of the schools, emergency responses, border and entry control measures, social isolation and nationwide social isolation measures, financial support, and other measures, so they reduce anxiety about diseases other than COVID-19 at that time [17].

We recognize the need to apply 5 ***K*** in the community, especially in medical facilities, reducing patient anxiety during follow-up visits and maintaining medication adherence. In addition to the partial impact of the COVID-19 epidemic, other factors (cost, drug burden, drug-use behavior) need to be considered more to achieve the goal of the treatment and to improve the medication adherence rates of patients with chronic diseases. Compliance with medication and scheduled visits during the COVID-19 pandemic is a matter of concern for patients during this period. Epidemic prevention measures at medical facilities are necessary to reduce the epidemic’s impact on patients’ follow-up visits and to limit disease transmission.

Research on a larger scale or in other regions of Vietnam should be conducted to have more comprehensive and clearer survey data on the factors affecting chronic patient compliance during COVID-19. From there, health managers can devise measures and interventions to increase drug adherence for chronic patients in the context of a long-term epidemic based on those research results.

### 4.4. Strengths of the Study

The use of different rating scales is also why the research results differ from other studies. The sensitivity of the GMAS is 75% higher than that of the MMAS scale [18]. In addition, the GMAS can also assess the cost of treatment, while the MMAS scale does not address this issue but only focuses on the issue of drug-use behavior. In this study, we did not go into an in-depth analysis of drug-use behavior, such as with the MMAS scale, but we chose the GMAS to further assess the patient’s treatment costs. Because the COVID-19 epidemic has directly affected people’s income, it has indirectly affected drug adherence due to the treatment costs of patients with chronic diseases, specifically through our research. Specifically, the rate of non-compliance due to high cost (77.8%) was highest in the cardiovascular patients (81.4%; *p* < 0.001).

### 4.5. Weaknesses of the Study

The treatment process outcome depends significantly on the patient’s psychology. When they are anxious or psychologically unstable, forgetting the doctor’s instructions or forgetting to take a few pills a day can happen. In this study, patients with cardiovascular and metabolic endocrine–metabolic diseases should be closely monitored to obtain the best treatment effect, and psychological issues should be paid more attention to. The questionnaire we used only asked about the patients’ diseases and the overall number of drugs they were taking, which placed some restrictions on our research. More particular medicine types and drug regimen complexity for diabetic patients (injectable insulins, etc.) and cardiovascular patients were not mentioned (e.g., through a routine aortic stent check). We recommend that there should be more in-depth psychological surveys during treatment in these patient groups and that researchers should explore the drug form that the patient had used more clearly to enrich the database of the factors affecting drug adherence, which would thereby lead to measure proposals to improve drug adherence and appropriate measures to maintain and enhance drug adherence rates in chronic patients during the COVID-19 pandemic. This study was conducted only in the southern provinces; there may be differences in patient characteristics and the local epidemic situation between different regions of Vietnam.

## 5. Conclusions

Medication adherence is an issue that needs to be taken care of in treatment to bring the best effects to the patient. The study results showed that financial factors affected drug adherence in all three groups of patients. There should be better health insurance policies, and doctors should consider giving lower-cost prescriptions to outpatients. The COVID-19 pandemic affects attitudes and acts as a barrier to medication adherence in patients with chronic diseases. It should be mentioned that people with chronic diseases are more at risk of developing severe symptoms and dying from COVID-19. Educating the public better on medication adherence during treatment will reduce severe symptoms and death from COVID-19 (either directly due to COVID-19 worsening the condition, or indirectly due to the psychological effects of COVID-19, the attitude of not going to the hospital for regular appointments, and/or giving up treatment leading to a chronic illness with unmanaged progress). In addition, regulatory agencies must take care of people’s welfare and maintain current policies to protect them from COVID-19 infection in the community and health facilities.

## Figures and Tables

**Table 1:** The demographics of the patients (*n* = 1444).

Characteristics	Total *n* (%) 1444 (100)	Group			*p* *
		Cardiovascular *n* (%) 694 (48.1)	Endocrine–Metabolic *n* (%) 405 (28.0)	Both of Them *n* (%) 345 (23.9)	
Age (Mean ± standard deviation (years old): 61.85 ± 11.7)					
<60 years old	578 (40.0)	276 (39.8)	187 (46.2)	115 (33.3)	<0.001
≥60 years old	866 (60.0)	418 (60.2)	218 (53.8)	230 (66.7)	
Gender					
Males	628 (43.5)	310 (44.7)	178 (44.0)	140 (40.6)	0.352
Female	816 (56.5)	384 (55.3)	227 (56.0)	205 (59.4)	
Living place					
Rural areas	783 (54.2)	374 (53.9)	221 (54.6)	188 (54.5)	0.984
Urban areas	661 (45.8)	320 (46.1)	184 (45.4)	157 (45.5)	
Employment					
Employed ^a^	223 (15.4)	111 (16.0)	57 (14.1)	55 (15.9)	0.474
Unemployed ^b^	1221 (84.6)	583 (84.0)	348 (85.9)	290 (84.1)	
Level of education					
Illiterate/Elementary	513 (35.5)	244 (35.2)	145 (35.8)	124 (35.9)	0.968
High school or higher	931 (64.5)	450 (64.8)	260 (64.2)	221 (64.1)	
Monthly income					
≤5 million VND	838 (58.0)	401 (57.8)	230 (56.8)	207 (60.0)	0.374
>5 million VND	606 (42.0)	293 (42.2)	175 (43.2)	138 (40.0)	
Duration of disease (Mean ± standard deviation (years): 8.07 ± 6.19)					
≤5 years	625 (43.3)	316 (45.5)	202 (49.9)	107 (31.0)	<0.001
>5 years	819 (56.7)	378 (54.5)	203 (50.1)	238 (69.0)	
Duration of treatment (Mean ± standard deviation (years): 7.86 ± 6.13)					
≤5 years	655 (45.4)	332 (47.8)	209 (51.6)	114 (33.0)	<0.001
>5 years	789 (54.6)	362 (52.2)	196 (48.4)	231 (67.0)	

^a^ Being paid to work for a company or organization. ^b^ Retirement, running own business, etc., without being paid to work for a company or organization. * *p*: comparing the difference between endocrine–metabolic disease and patients with both diseases (cardiovascular and endocrine–metabolic).

**Table 2:** Adherence in each disease group attended to the GMAS.

Group of Diseases	High Adherence (30–33) *n* (%)	Good Adherence (27–29) *n* (%)	Partial Adherence (17–26) *n* (%)	Low Adherence (11–16) *n* (%)	Poor Adherence (0–11) *n* (%)
Cardiovascular	155 (38.3)	96 (23.7)	136 (33.6)	15 (3.7)	3 (0.7)
Endocrine–metabolic	264 (38)	160 (23.1)	253 (36.5)	17 (2.4)	0 (0)
Both of them	115 (33.3)	77 (22.3)	121 (35.1)	22 (6.4)	10 (2.9)
Total	534 (37.0)	333 (23.1)	510 (35.3)	54 (3.7)	13 (0.9)

**Table 3:** Adherence features according to the GMAS in each disease group.

Medication Adherence		Medication Adherence		OR (95% CI)	*p* *
		Adherence *n* (%)	Non-Adherence *n* (%)		
Non-adherence due to patient behavior	Cardiovascular	471 (67.9)	223 (32.1)	1	-
	Endocrine–metabolic	299 (73.8)	106 (26.2)	1.336 (1.017–1.755)	0.037
	Both of them	211 (61.2)	134 (38.8)	0.746 (0.570–0.975)	0.032
	Total	981 (67.9)	463 (32.1)		
Non-adherence due to additional disease and pill burden	Cardiovascular	571 (82.3)	123 (17.7)	1	-
	Endocrine–metabolic	327 (80.7)	78 (19.3)	0.903 (0.659–1.237)	0.525
	Both of them	266 (77.1)	79 (22.9)	0.725 (0.528–0.997)	0.047
	Total	1164 (80.6)	280 (19.4)		
Non-adherence due to financial constraints	Cardiovascular	129 (18.6)	565 (81.4)	1	-
	Endocrine–metabolic	92 (22.7)	313 (77.3)	1.287 (0.952–1.739)	0.1
	Both of them	99 (28.7)	246 (71.3)	1.763 (1.304–2.383)	<0.001
	Total	320 (22.2)	1124 (77.8)		

* *p*: comparing the difference between endocrine–metabolic patients and patients of both diseases (cardiovascular and metabolism).

**Table 4:** COVID-19 prevention measures by 5 ***K*** message and the relationship between measures to prevent COVID-19 and each disease group.

Group of Disease	Mean ± Standard Deviation (Measures)	>1 Measure *n* (%)	≤1 Measure *n* (%)	COVID-19 Prevention Measures				
				Wearing Facemask *n* (%)	Disinfection *n* (%)	Distance *n* (%)	No Gathering *n* (%)	Health Declaration *n* (%)
Cardiovascular	1.91 ± 1.188	383 (55.2)	311 (44.8)	383 (94.6)	178 (44.0)	82 (20.2)	57 (14.1)	65 (16.0)
Endocrine–metabolic	1.89 ± 1.229	207 (51.1)	198 (48.9)	658 (94.8)	326 (7.0)	133 (19.2)	93 (13.4)	118 (17.0)
Both of them	1.97 ± 1.152	203 (58.8)	142 (41.2)	334 (96.8)	164 (47.5)	62 (18.0)	54 (15.7)	67 (19.4)
*Total*	1.92 ± 1.191	793 (54.9)	651 (45.1)	1375 (95.2)	668 (46.3)	277 (19.2)	204 (14.1)	250 (17.3)

**Table 5:** The relationship between patient characteristics and medication adherence.

Characteristics		Medication Adherence		Univariable Logistic Regression		Multivariable Logistic Regression	
		Adherence *n* (%)	Non-Adherence *n* (%)	OR (95% CI)	*p*	OR (95% CI)	*p*
Age	≥60 years	381 (65.9)	197 (34.1)	1.512 (1.216–1.881)	<0.001	1.264 (1.002–1.593)	0.048
	<60 years	486 (56.1)	380 (43.9)	1	-	1	-
Gender	Males	387 (61.6)	241 (38.4)	1.124 (0.909–1.391)	0.281	-	-
	Female	480 (58.8)	336 (41.2)	1	-	-	-
Living place	Rural areas	437 (66.1)	224 (33.9)	1.602 (1.293–1.984)	<0.001	1.377 (1.096–1.729)	0.006
	Urban areas	430 (54.9)	353 (45.1)	1	-	1	-
Employment	Employed ^a^	163 (73.1)	60 (26.9)	1.995 (1.453–2.739)	<0.001	1.607 (1.146–2.253)	0.006
	Unemployed ^b^	704 (57.7)	517 (42.3)	1	-	1	-
Level of education	High school or higher	598 (64.2)	333 (35.8)	1.629 (1.308–2.028)	<0.001	-	-
	Illiterate/Elementary	269 (52.4)	244 (47.6)	1	-	-	-
Duration of disease	≤5 years	401 (64.2)	224 (35.8)	1.356 (1.095–1.680)	0.005	-	-
	>5 years	466 (56.9)	353 (43.1)	1	-	-	-
Duration of treatment	≤5 years	414 (63.2)	241 (36.8)	1.274 (1.030–1.576)	0.025	-	-
	>5 years	453 (57.4)	336 (42.6)	1	-	-	-
Group of disease	Cardiovascular	424 (61.1)	270 (38.9)	1.251 (0.963–1.625)	0.093	-	-
	Endocrine–metabolic	251 (62)	154 (38)	1.299 (0.970–1.740)	0.076	-	-
	Both of them	192 (55.7)	153 (44.3)	1	-	-	-
Comorbidities	0–1	625 (62.4)	377 (37.6)	1.370 (1.092–1.719)	0.006	1.363 (1.074–1.729)	<0.001
	≥2	242 (54.8)	200 (45.2)	1	-	1	-
COVID-19 prevention measures	5	103 (88)	14 (12)	6.445 (3.612–11.502)	<0.001	5.826 (3.237–10.486)	<0.001
	2–4	417 (61.7)	259 (38.3)	1.411 (1.134–1.755)	0.002	1.315 (1.051–1.647)	<0.001
	≤1	347 (53.3)	304 (46.7)	1	-	1	-
COVID-19 pandemic obstructed treatment follow-up visits	No	444 (66.4)	225 (33.6)	1.642(1.326–2.034)	<0.001	1.594 (1.278–1.989)	<0.001
	Yes	423 (54.6)	352 (45.4)	1	-	1	-
Feeling worried during visits	No	271 (66.1)	139 (33.9)	1.433 (1.128–1.819)	0.003	-	-
	Yes	596 (57.6)	438 (42.4)	1	-	-	-

^a^ Being paid to work for a company or organization, ^b^ retirement, running own business, etc., without being paid.

## Data Availability

Data are contained within the article. Data sharing is not applicable to this article.
